# Hospital and Institutional News

**Published:** 1915-05-01

**Authors:** 


### HOSPITAL AND INSTITUTIONAL NEWS.

### THE KING AT THE GREAT NORTHERN HOSPITAL.

An unexpected visit to the Great Northern Central Hospital, Holloway, was paid by the King and Queen on Thursday last week. They were received at the institution by Sir Robert Burnet, M.D., member of the general committee; Mr. C. Walton Sawbridge, chairman of the house committee; and the secretary, Mr. Gilbert Panter, as representing the administration. The medical staff was represented by the senior physician, Dr. Alexander Morison; the resident medical officer, Dr. Maurice Horan; and the matron, Miss Bird, represented the nurses. The King and Queen proceeded immediately to the two wards which have been set apart for military patients and entered into conversation with each of the seventy men, interesting themselves in the actions in which they had fought. Queen Mary addressed with special interest one of the men of her own regiment, the 18th (Queen Mary's Own) Hussars. It was explained that in addition to the patients in the hospital, thirty more are recuperating at the Reckitt Convalescent Home, Clacton-on-Sea, which is now being used as an auxiliary hospital. After leaving the soldiers' wards the King and Queen visited the children's ward, which had twenty occupants, in whom the Royal visitors showed their interest. Then, before leaving the institution, those of the soldiers who were approaching convalescence gave three cheers so hearty as to be taken up by the crowd outside, which had assembled in numbers. Elsewhere in this issue we draw attention to the immense amount of help which has resulted from gifts in kind to various hospitals since the outbreak of war, and we trust that all hospitals which, like the Great Northern, are actively attending to the wounded may receive continued support in this respect as well as from gifts in money.

### THE WOMAN HOSPITAL ORDERLY.

We have already recorded the advent of the woman hospital porter, and the success which, so far, has attended her labours at one county hospital. Another scheme, this time more in the nature of an organised addition to voluntary aid work, has now been inaugurated in Liverpool for a corps of women hospital orderlies. The corps has sought recognition at the hands of the Joint Committee of the British Red Cross Society and the Order of St. John, and is designed "to provide helpers for all purposes in the temporary military hospitals in and around Liverpool, or, if necessary, elsewhere." It may be further explained that the women orderlies are intended to live either in their own homes or in the hospitals, as may be found convenient, and to take duty for a month at a time. It is suggested that they should do such work as helping with the cooking and washing, keeping stores, waiting on nurses, and making themselves generally useful. The promoters believe that all the early volunteers will pay for their own uniforms and expect no salary. So far as work goes, the duties of the woman hospital orderly seem very closely to resemble those of the so-called part-time probationer, but the new name has at least the advantage of making plain at the outset that nursing, in the professional sense, is not required at the hands of volunteers. No doubt after nine months of war the necessary limitation of their duties has impressed itself upon all the early probationers who have had sufficient grit to stick to their posts, but at the same time for women the name "hospital orderly" is less likely is mislead than that of "probationer," which has professional associations.

### KING ALBERT'S HOSPITAL FOR CONVALESCENT BELGIAN SOLDIERS.

The Bermondsey Board of Guardians in the early stages of the war, when large numbers of Belgian refugees came to this country, placed at the disposal of the authorities their institution in Tanner Street, and the manner in which the medical staff as well as the officials entered into the care of the unfortunate persons compelled to seek a shelter earned for them the commendation of the Government authorities as well as of the Belgian Embassy. Immediately after the last allocation of the refugees took place from the institution, arrangements were made for it to be used as a reception depôt and distributing centre for réformés and convalescents of the Belgian Army. The institution has now changed its name to the King Albert's Hospital for Convalescent Belgian Soldiers. The clerk to the Guardians, Mr. Pitts Fenton, has been ably assisted by the members of the board in converting the institution into a comfortable hospital, the whole of the arrangements having been made under the direction and with the approval of the Local Government Board in conjunction with the Belgian Embassy.

### CENTRAL STORES FOR POOR-LAW INFIRMARIES.

The Local Government Board has replied to the suggestion made by the Southwark Board of Guardians, as reported in previous issues of The Hospital, that the authorities at Whitehall should establish central stores from which all the Poor-Law authorities in the Metropolitan area might draw their food, coal, and drug supplies. The Board state that they are unable to give effect to it at the present time. This is one of the replies of a vague character which have from time to time caused some uncertainty, and so it was in this case, for the clerk, questioned as to whether it meant that the Board approved of the suggestion, replied that he was unable to say. At all events it did not express disapproval, and this, no doubt, will incite those who have to provide large stores again to attempt to induce the Local Government Board to take action. Much has been written about the demands of the Guardians upon contractors, but at the present time, even when the most acute crisis has passed, the Metropolitan Boards of Guardians are practically at the mercy of their contractors, many of whom have proved unreasonably exacting. Attempts to meet the contractors in a fair spirit have often been met with increased demands, and this has led advocates of a central drug stores to urge that its establishment would do away with a great many of the abuses of the contracting system which have been apparent during the past few months.

### AN EMERGENCY MEASURE.

From the articles with reference to shock and traumatic neurosis which have appeared in our columns it has been made plain that the state of the law with reference to mental disease and its treatment in England was unsatisfactory. A Home Office Bill was, therefore, introduced last week "to facilitate the early treatment of mental disorder of recent origin arising from wounds, shock, and other causes." It is an emergency measure designed to be in force for the duration of the war and for six months afterwards, except as regards persons then under treatment. As our readers will recall, at the present time the law does not permit nursing homes to receive patients suffering from mental neurosis; such patients must be certified under the Lunacy Acts, even though the ailment be a temporary one. The new Bill would remove the necessity for formal certification in cases in which the mental disease arises from the specified causes of wounds and shock, as quoted above. The measure would, in this respect, assimilate the English to the Scottish law. A memorandum prefaces the Bill, and points out that its passage would enable a man who, in the service of his country, has suffered a nervous breakdown, to accept treatment without being certified, "but only for a period not exceeding six months and under conditions which will provide security against misuse." It is important to remember that the Scotch law has enabled institutions to be established for the benefit of those mental cases which may be expected to respond to treatment in a short time, while dispensing with immediate certification. There is thus provided an existing example of the results obtainable under a law modified on the lines suggested in the Bill. The institutions would not be those associated with the administrative care of lunacy, and the period of residence would be limited to six months.

### FROM HOSPITAL FOREMAN TO CHAIRMAN OF THE GUARDIANS.

At a recent meeting of the Leicester Board of Guardians Mr. Walter Smith was elected chairman for the ensuing year. The appointment is of interest by reason of the fact that some twelve years ago the new chairman occupied the position of foreman of the works at the Leicester Royal Infirmary—a position he resigned to enter upon a business career as a builder and contractor. Mr. Smith has devoted a great deal of time to Poor-Law administration and to the work of the various committees, especially the relief and assessment. His long experience of building and his extensive knowledge of farm and garden work will serve him well in the important post he now fills, and should make his advice and counsel of special value.

### LEICESTER AS A HOSPITAL CENTRE.

Rapid progress is reported from the base hospital at Leicester in the temporary buildings which are now being erected to increase the accommodation of this hospital to more than 1,000 beds. Our readers will remember that the North Evington Infirmary was recently taken over by the military authorities for the accommodation of 600 patients, and, with a view to still further provision, the governors of the Leicester Royal Infirmary have been asked by the War Office to place, in addition to 60 beds already used, a further 100 beds at the disposal of the military authorities, a request which has been complied with. Provision is thus made for the reception and surgical treatment of some 1,800 wounded soldiers in Leicester, in addition to considerable accommodation for convalescent patients in V.A.D. hospitals.

### AN EXTRAORDINARILY BELATED REPORT.

In recent years various questions relating to the public health have been inquired into by Royal Commissions, but few of them have covered such a large ground as the problem of the disposal of sewage, on which a Commission, appointed over sixteen years ago, has now issued its final Report! In the interval of years since the Commission was appointed ten Reports have been issued, the whole forming an extraordinarily voluminous mass of literature on an enormously important but very depressing subject. As its final contribution this Royal Commission has published a Blue Book dealing with the disposal of liquid wastes from manufacturing processes and the disposal of liquid and solid refuse in rural areas. The unhealthy state of rivers in industrial districts is largely due to waste liquids which are discharged into the rivers from the factories; if these liquids were purified before discharge the rivers might be at least passably agreeable to the eye and not offensive to the nose, but unfortunately the process of absolute purification is too costly and often impracticable. Briefly, what the Commission recommends is that a certain degree of purification should be insisted upon, and if this were done the state of the rivers and incidentally the health of the neighbouring population would, no doubt, be improved. With regard to the disposal of refuse in rural areas the Commissioners recommend an extension of the water-carriage system wherever possible, although dry closets may be used satisfactorily under proper supervision in places where a water supply is not laid on to the houses.

### A COTTAGE HOSPITAL AND THE WAR OFFICE.

A rather curious position of affairs, partly due to the war, has arisen in connection with the Woolwich and Plumstead Cottage Hospital at Shooter's Hill, which was built as a Jubilee memorial in 1887 and opened in 1890, on a site which belongs to the War Office. The building, which was designed by Mr. J. O. Cook, accommodates twelve beds and two cots, but now that the governors are anxious to extend the hospital to meet the needs of the growing population they are brought up against the refusal of the War Office to sell any land to the hospital owing to the possibility of the land being wanted for further military purposes. There has seemed, then, no alternative to building anew on a fresh site if the present inadequate accommodation is to be replaced by a larger hospital. But to decide on a new site is not an easy matter topographically, and though there is much to be said in favour of rebuilding as against additions, this argument has more weight in the case of larger institutions, and only affects cottage hospitals with full force when they began existence in adapted premises.

### LEWISHAM INFIRMARY AS A MILITARY HOSPITAL.

The Army Council have taken over the Lewisham Infirmary and the adjoining workhouse as a hospital for wounded and sick soldiers. It is contemplated that the authorities will be able to provide 1,000 beds in the institutions, and the delightful premises on the Lewisham High Street should be appreciated by the soldiers, as they have been by the inmates. Some pathetic scenes were witnessed when the patients were removed to the institutions in neighbouring parishes, where accommodation had been found for them. Some of the chronic invalids had been in the infirmary for over ten years, indeed a few for close on twenty years, and their removal from what to them had become a permanent home was the cause of much distress and grief. The staff at Lewisham have always taken a personal interest in their charges, and Dr. Toogood, the medical superintendent, has been an invigorating stimulant to the patients. Hence their grief at a short severance from associations that were to them of a most personal character. In other infirmaries it is to be hoped they will meet with the same kindly personal interest as they have experienced at Lewisham under Dr. Toogood and his staff.

### AN ATMOSPHERE OF ECONOMIC RESEARCH.

When the word "research" is employed in hospital circles it is used exclusively to cover the investigations of medical science. But this is really becoming quite an arbitrary limitation, since, as was well said by an able hospital manager only this month, anyone who lived as he had done for several years in an atmosphere of economic research, knew that it became harder every year to make further discoveries. This apt description of the labours of an up-to-date keen committeeman was the more remarkable in that it was used in a speech the object of which was to disclaim any credit for the surprising fact that the expenditure on provisions for the year 1914 showed a decrease of £42 notwithstanding the rise in prices and the increased number of staff and patients which the institution had had to feed during the last quarter. What is the explanation? It was solely due, the speaker explained, to the enormous quantity of gifts in kind and provisions of every description which had come from all parts of the world, including the Colonies. This is the most striking testimony we have seen to the importance of these gifts, and to the place which an endeavour to maintain them should hold in economic research.

### WOMAN ON HER OWN.

The immense scope which the war has given to the employment of women must not blind our eyes to the fact that some of the work on which they have been engaged may perhaps prove unsuitable as a permanent occupation—for instance, that of railway porter. It is therefore to be hoped that the present practical opportunity of gleaning from experience what new occupations are shown to be well within their capacities—and there must be many—should not be lost. On this ground the exhibition known as "Women and their Work," which is being organised by the *Daily Express* at the Royal Horticultural Hall, Westminster, to be opened to-day, may prove of value. The exhibition, which has the double intention of epitomising every phase of women's work from agriculture to jewellery, and of providing women with ideas for new means of earning a livelihood, is of interest to institutional readers from the part which nursing and hospital work must inevitably play in it. Indeed, we understand there will be a special section devoted to this subject, in which they have been acknowledged as essential participators, and one of the exhibits will show a ward of the Middlesex Hospital, while another will comprise an exhibition of invalid kitchens by Lady Muriel Paget. There will be lectures on hygiene; while Dr. Elizabeth Sloan Chesser is to lecture on "War's Effect on Marriage and Women's Work." Our account last week of the county hospital at which women have been engaged as porters serves as a reminder how directly interested hospital managers are in the new extension of "women's sphere," which the war has done more to enlarge than all the theorisers, speakers, and militants, from John Stuart Mill to Mrs. Gilman.

### THE INDIFFERENT TESTATOR.

The indifference of testators to the exact names and designations of the institutions which they wish to benefit is regularly the occasion of actions in the Courts, and a recent case before the Lancashire Chancery Court illustrates this indifference very markedly. A petition was presented to the Court by the trustees of the will of the late Frederick Marshall Kenderdine for directions as to the payment of two legacies and certain shares in the residue of the estate to two Manchester institutions. One was a legacy of £500 to St. Mary's Hospital, Quay Street, and the other of £200 to the Southern Hospital for the Diseases of Women and Children. At the date of the will, it was pointed out, the two institutions were separate, but by an order of the Charity Commissioners made in 1906 a scheme was approved under which it was provided that the two charities should be administered as one under the designation "The St. Mary's Hospitals, Manchester." The question therefore was whether the St. Mary's Hospitals, Manchester, was entitled to receive the two legacies of £500 and £200 and any further moneys that might fall in under the will. After hearing arguments on behalf of the hospitals and of other interested parties it was decided that both legacies were payable to the St. Mary's Hospitals, Manchester. It is to be noted that at the date of the testator's death, which occurred in June 1914, the institutions had been amalgamated for eight years, and yet during that time it apparently never occurred to him to alter the words of his will so as to guarantee that the institutions which he favoured should benefit. It is only fair to add, however, that a large number of charitable institutions which had also benefited under the present will were not concerned in the above petition.

### A PROJECTED COTTAGE HOSPITAL FOR COALVILLE.

We recently reported in our columns a generous offer by Mr. James Wootton of £1,000 in memory of his wife and son, to establish a cottage hospital in this populous centre of Leicestershire. It was proposed to expend £2,500 on the building of a hospital of twelve beds, but the response to the appeal for funds was limited, and at a public meeting of the town a resolution was passed accepting Mr. Wootton's generous offer, but asking him, in view of the critical times and financial uncertainty, to extend the time limit of his gift to the end of the year. We hope that Mr. Wootton will see his way to agree to the modification suggested, because we feel it will be a great advantage to the town and district to have a small but well-equipped hospital established there. As, however, the Leicester Royal Infirmary has for years borne the burden of providing hospital accommodation for the town and district round Coalville, and has been compelled, largely by the increasing pressure of the demands upon its accommodation, to spend a sum of £120,000 on hospital extensions and improvements, it ought to be urged upon the residents of Coalville that the annual cost of maintenance of the proposed cottage hospital should be raised independently of and additional to the sum now collected and sent to the County Hospital. It would obviously be unfair to the Royal Infirmary to deprive it of the income which has been built up by its methods of organisation, and we are glad to see that this view received definite recognition at the meeting from Mr. Walter Lindley, the presiding chairman.

### THE BED PROBLEM AT DERBY.

As typical of the amount of war work being performed by the county hospitals the case of the Derbyshire Royal Infirmary may be quoted, where at the close of the half-year 289 wounded men have been received, of whom 271 passed to Red Cross hospitals or received sick furlough, one died, and seventeen remained. April was made noteworthy by the recent opening of the Markham Pavilion, a wooden structure in the hospital grounds built through the generosity of Mr. C. P. Markham, of Chesterfield, to provide further accommodation for the wounded. By its means will be mitigated the difficulty created through the varying length of stay of different patients, which causes empty beds. These cannot be used for civilians in case a new convoy arrives, but the new pavilion can be filled while keeping a reserve of 100 beds for newly-arrived wounded.

### MONTENEGRO AND SANITATION.

Typhus and typhoid fever are prevalent in Montenegro, and, as mentioned in "Round the World," a woman doctor has been appointed to undertake the work of prevention in this centre. The Wounded Allies' Relief Committee are sending Dr. Isabel Ormiston, a graduate of Sydney University, to Montenegro to aid in organising a proper medical service in view of the epidemics. Dr. Ormiston, who is understood to have made a special study of sanitation, for the past three years has held the post of Chief Medical Inspector of Schools to the Tasmanian Government. In this connection it may be added that the British Farmers' Red Cross Fund is preparing a second hospital to follow the first, owing to the spread of typhus in Serbia.

### THE SIGHT OF THE BLIND.

Among the permanent disablements resulting from wounds and injuries during active service none is more crushing than that of blindness, and our readers will turn with interest to the letter from Mr. C. Arthur Pearson, which we publish this week, with reference to the valuable work which is being done in making them as far as possible independent at St. Dunstan's Hostel, Regent's Park. Aptly enough the King and Queen visited St. Dunstan's on Monday, and there saw with their own eyes the blinded sailors and soldiers busy at their adopted occupations of carpentry, basket and boot making, mat-weaving, and, indeed, typewriting. It is needless here to enlarge on how the ingenuity of mankind can make four senses do the work of five by adaptation, but it is interesting to recall that the sense which is most relied on—namely, of course, touch—is fundamentally the one sense of which tasting, hearing, and so on are but the variants. So at St. Dunstan's men were observed by the King and Queen reading in Braille, writing from dictation, and, as noted above, using the typewriter, both of the ordinary or Braille design. Mr. Pearson, who showed the King and Queen round, received expressions of their gratification at the success which is attending the work and at the conditions, pleasant as possible, under which it is conducted. Such human ingenuity as we see here employed must serve as a set-off to our consciences for the ingenuities for mischief and destruction which have caused modern warfare to be the machine-made inferno which it is.

### WORCESTER GENERAL INFIRMARY.

Mr. Shrewsbury Smith has been re-elected as chairman of Worcester General Infirmary. Mr. S. T. Harris is the new vice-chairman. The spirit of interest shown by families associated with the hospital's work was seen at the monthly meeting, when the secretary announced that the children of the late Mr. G. G. Hyde, formerly hon. surgeon to the infirmary, had sent £1,000 for the endowment of a bed in a surgical ward in memory of their father. The committee gratefully accepted the offer, and decided that a donation of £1,000 carried with it the privilege of naming a bed, and that a gift of £500 entitled the donor to the same privilege respecting a child's cot. The retiring vice-chairman, Mr. R. Bruce Ward, was cordially thanked for his services during the past two years. In replying, Mr. Ward said he agreed that the office should go round. The question of rewarding the matron and a sister for special work in connection with the wounded is being considered.

### THE LONDON SCHOOL MEDICAL SERVICE.

It is satisfactory to learn that this year, at any rate, the London school medical service gives complete satisfaction to the Board of Education, which has criticised it in some previous years. The London County Council have just been informed by the Board that it has decided to make a grant of £36,149 towards the expense of the medical service, and the letter adds: "The Board are glad on this occasion to recognise by the payment of grant at the maximum rate the steps taken by the authority to remedy the defects in their school medical service to which attention was drawn as the result of the last inspection of the work by the Board's medical officers." It is pointed out, however, that the assessment will be subject to annual revision, which will be made in the light of improvements and developments in the work.

### THE SALE OF NARCOTIC DRUGS.

Two important cases were heard at the Mansion House Police Court last week, in which the question involved was the sale of opium. The defendants were produce brokers in a very large way of business, and the offence alleged against them was that they had sold to a person unknown to them a quantity of opium without making such record of the transaction as is required by the Pharmacy Acts, and without labelling the packets with the word "Poison." In each case the amount of the purchase was 10 lb.; this to a doctor or a chemist must seem an enormous quantity, but to a drug broker accustomed to dealing in colossal quantities the amount would probably appear small. Both defendants were fined the full penalty allowed by the Pharmacy Acts, and an undertaking was given that the offence would not be repeated. The important point is that while the sale of opium in small quantities is ordinarily subjected to severe restrictions, it was possible for a man out of the street to walk into a broker's office and come out with enough opium to poison a large proportion of the population of the City of London. It is not in the public interest that this sort of thing should be possible, and the Pharmaceutical Society performed a service to the community in bringing these proceedings.

### THE WAR OFFICE AND THE PRIVATE HOUSE.

On the outbreak of war we commented on the recklessness with which private houses were being offered and, in some cases, accepted for the treatment of the wounded. Time has served to sift these out, but that a large number of persons are still letting their eagerness get the better of their judgment is apparent from a pronouncement which the War Office has seen fit to make upon the matter. Owing, says this statement, to the establishment on a large scale of military hospitals for the reception and treatment both of sick and wounded soldiers and convalescents, it is not proposed to accept any further offers of private houses for auxiliary hospitals or convalescent homes. The logic of facts has no doubt forced this decision upon the authorities, and it is a wise plan to rely mainly on institutional buildings for hospital purposes, though even here, as the case at Leicester well illustrated, schools and local government offices are much to be avoided wherever, by co-operation of existing hospitals and infirmaries, and by the erection of suitable temporary buildings on open grounds, premises, new or old, are obtainable which have been designed from the first for the reception of the sick.

### THE FATE OF THE CIRCULAR.

Not long ago we illustrated a highly ingenious appeal card on which the main features were a waste-paper basket (on the left), and a reproduction of a cheque (on the right), with the words "Oh, not this!"—"But—This" written over each of them. Such a circular may have a reward, appealing at once to the public's sense of humour, which when aroused often leads to a gift of money; but that a circular must have very special features, and an individual character of its own, is becoming realised, but still is not realised enough. That, at least, is the moral which we draw from the fate which has attended a circular issued by the Edinburgh Royal Infirmary. Before detailing the result it should, perhaps, be explained that we have not seen the actual document, and so do not know its merits or defects; judgment is based upon the published results alone. What were they? In connection with a special appeal there were issued on March 12, 14,702 copies of a circular. By April 10, 646 responses had been received—that is to say, for every twenty-three circulars issued, one reply was received. No one can pretend that that is a satisfactory result, but the figures are telling as showing the degree of failure with which a circular may meet. As a rule, circulars should be only the second string. The main one should be a carefully organised meeting or series of meetings, with specially chosen speakers, ending with a plan of campaign. A circular gives its recipient no trouble. The line of least resistance is to throw it away. But a meeting, followed by a collector; anything in which the individual members can take part; that is the road to interesting them. Indeed, the circular also gives too little trouble to those who issue it, another defect quite as grave as the first. But the circular at Edinburgh will have been useful if its figures are borne in mind; if it could not be a success, it may yet be a warning, and as such is worth quoting here.

### CHAIRMAN OF WEST HAM HOSPITAL RESIGNS.

Mr. T. A. Cook, who has been connected with West Ham Hospital since 1895, has resigned his position as chairman of the institution, and at the last meeting of the management committee the following resolution was unanimously passed: "That a vote of very sincere thanks be accorded to Mr. Cook for his untiring efforts on behalf of the hospital during the many years he has occupied the chair, and for the great and valued services he has rendered to the institution; and that the hope be expressed that although Mr. Cook has resigned his position as chairman of the committee he nevertheless will see his way to continue to take an active interest in the welfare of the hospital." Mr. C. E. Leo Lyle, the president of the institution, is, we understand, temporarily acting as chairman of the committee. There is no doubt that Mr. Cook's resignation will be a very real loss to the hospital, for he was an enthusiastic voluntary worker, always on the alert for new ways of extending the hospital's usefulness, and for increasing public interest in its welfare and support.

### THE HOME-MADE RESPIRATOR.

It is perhaps surprising that the resources of science generally for destruction other than by explosives have been less used than is actually the case in modern warfare, although the recent use of asphyxiating gases by the Germans, in contravention of the terms of the Hague Convention, show what malign possibilities lie in this direction. The new agent necessitates the use of simple medical precautions, and, as a matter of history, it is valuable to record the following appeal from the War Office: "As a protection against the asphyxiating gases being used as a weapon of warfare by the Germans, supplies of one or both of the following types of respirator are required by the troops at the Front. Either can be made easily in any household." The first type is described as a face-piece to cover the mouth and nostrils, formed by an oblong pad of bleached absorbent cotton-wool about 5¼ by 3 by ¾ in., covered with three layers of bleached cotton gauze, fitted with a band to fasten round the head, consisting of a piece of ½ in. cotton elastic 16 in. long, attached to the narrow end of the face pad, so as to form a loop with the pad. The second type is a piece of double stockingette, 9½ in. long, 3½ in. wide in the centre, gradually diminishing in width to 2½ in. at each end, with a large piece of thick plaited worsted about 5 in. long attached at each end, so as to form a loop to pass over the ear. These respirators should be sent in packages of not fewer than 100 to the Chief Ordnance Officer, Royal Army Clothing Department, Pimlico. Hospitals which depend on voluntary service will note with interest this general appeal for home-made appliances to the nation, and also how medical measures are now shown to be as important in repelling hostile attacks as they have been in preventing epidemic disease behind the lines. May they prove equally successful!

### COLOUR IN THE ANNUAL REPORT.

The successful attempts of recent years to brighten the annual report by making it more readable in itself, by lightening its statistics with illustrations, and by generally making the cover and format as attractive as possible have taken comparatively rapid strides, and have justified the increased cost incurred. The process may still be continued with advantage, and we note for its enterprise the remarkable features which have been incorporated in the report of the City of London Hospital for Diseases of the Chest, just issued. The cover is printed in colours, chocolate, blue, and green; and in the centre is a vignette of the institution, also in colours. Just inside the cover appears a four-fold sheet, which reveals itself on unravelling as a reproduction of a scroll commemorative of the foundation-stone-laying of the hospital, duly signed by the Prince Consort and others. Facing page 2 is another reproduction in colours of the institution, which, while inevitably somewhat brightening the colours of bricks and trees in London, is yet very effective, and does after all impress on the reader the relation of the hospital to Victoria Park. Further on is another coloured plate representing the patients engaged in light manual work in the grounds, but this, perhaps because, being a garden picture, the whole is in green, is not as satisfactory as the first. There are also several ordinary photographs, and the hospital's committee and Mr. Watts, the secretary, are to be congratulated on their enterprise in introducing colour into the annual report. By the way, might not the fine doors and old brasswork in the hospital provide suitable subjects for small illustrations, or drawings inset in the text, in a future report?

### THIS WEEK'S DRUG MARKET.

The character of the drug market is unchanged; that is to say, the demand is active and prices continue in their upward tendency. But there have been few, if any, of those remarkable advances in price which from time to time have been remarked upon during the past few months. Quotations for many of the synthetic drugs have now reached such extremely high figures that buyers are exercising great caution. Recently supplies have arrived of some of the much-wanted drugs, and further supplies are understood to be *en route* from neutral countries; but it is questionable whether they will be in sufficient quantity to bring down prices to any material extent. The value of cod-liver oil is not so firmly maintained, and buyers do not seem eager to come forward. Tartaric acid and citric acid have an upward tendency in price, and as the season for that class of beverage in the manufacture of which these substances are largely employed is approaching it seems almost certain that prices will advance. There is a shortage of balsam of Peru, and very high prices are asked. There is no change in the position of cocaine, and its future is very uncertain.

